# Silver and Gold Compounds as Antibiotics

**DOI:** 10.3390/antibiotics13090850

**Published:** 2024-09-05

**Authors:** Christina N. Banti, Sotiris K. Hadjikakou

**Affiliations:** Laboratory of Biological Inorganic Chemistry, University of Ioannina, 45110 Ioannina, Greece; cbanti@uoi.gr

This Special Issue entitled “Silver and Gold Compounds as Antibiotics” covers a selection of recent research and review articles focused on biological inorganic chemistry. This Special Issue offers a comprehensive summary of the latest advancements and emerging trends in the rapidly evolving field of the role of silver and gold compounds as antibiotics. The insights provided in this Special Issue will enhance readers' understanding of antimicrobial metal compounds.

Infectious diseases remain a significant global health threat, being among the most difficult challenges in modern medicine [1]. With the growing resistance of bacteria to conventional antibiotics, the need to discover and develop new antimicrobial agents has become increasingly urgent [1]. Silver and gold salts have been used for centuries to combat microbial infections, and the development of new silver and gold compounds is now crucial in overcoming the limitations of existing antibiotics and preventing the emergence of multidrug-resistant bacteria [2,3].

This Special Issue brings together a wide range of articles examining the potential of silver and gold compounds in antimicrobial applications. It is composed by eleven articles or reviews, including the following:

- Judita Puišo et al. in “Antimicrobial Properties of Newly Developed Silver-Enriched Red Onion–Polymer Composites” show that biogenic silver nanoparticles, produced in a cyanobacterial culture in various sizes, effectively combat a range of local fruit pathogens, highlighting their potential as versatile, eco-friendly biocontrol agents that support food security and sustainability [4].
- Munirah F. Aldayel et al. in “Differential Antimicrobial Effect of Three-Sized Biogenic Silver Nanoparticles as Broad-Spectrum Antibacterial Agents against Plant Pathogens” report the synthesis and characterization of seven different silver nanoparticles (AgNPs) from various fungi isolated in Brazil, demonstrating their broad-spectrum antifungal activity against both pathogenic yeasts and agricultural phytopathogens, with potential for diverse applications due to their effective and non-specific action [5].
- Luiz Gustavo Ribeiro et al. in “Antifungal Activity of Mycogenic Silver Nanoparticles on Clinical Yeasts and Phytopathogens” demonstrate the development of antimicrobial 3D-printed collagen scaffolds with in situ synthesized silver nanoparticles (AgNPs) using UV irradiation, revealing that the method effectively controls the size and shape of AgNPs, enhances the thermal stability and swelling capacity of the scaffolds, and demonstrates high bactericidal activity against both Gram-negative and Gram-positive bacteria [6].
- Sofia Municoy et al. in “Development of 3D-Printed Collagen Scaffolds with In-Situ Synthesis of Silver Nanoparticles” investigate the antifungal and antiamoebic effects of silver nanorings (AgNRs) and compare them with other silver nanoparticles, revealing that AgNRs demonstrate notable antimicrobial activity against both fungi and amoebae, offering a promising alternative to traditional antifungal and antiamoebic therapies in the face of increasing drug resistance [7].
- Sara González-Fernández et al. in “A Promising Antifungal and Antiamoebic Effect of Silver Nanorings, a Novel Type of AgNP” investigate how varying the size of silver nanoparticles biosynthesized by Cyanothece-like cyanobacteria affects their antimicrobial efficacy, revealing that smaller nanoparticles are more effective against pathogenic bacteria, including MRSA and *Streptococcus* sp., with optimal size control achieved by adjusting precursor concentrations [8].
- Nermin A. El Semary et al. in “Multidrug-Resistant Bacterial Pathogens and Public Health: The Antimicrobial Effect of Cyanobacterial-Biosynthesized Silver Nanoparticles” demonstrate that cyanobacteria can biosynthesize silver nanoparticles that are active against pathogenic bacteria, and the size of silver nanoparticles can be controlled [9].
- Erika Alejandra Jardón-Romero et al. in “Antimicrobial Activity of Biogenic Silver Nanoparticles from *Syzygium aromaticum* against the Five Most Common Microorganisms in the Oral Cavity” found that biogenic silver nanoparticles synthesized using extracts of *Syzygium aromaticum* through green synthesis demonstrated effective antimicrobial activity against a range of microorganisms in oral cavities [10].
- Elvira Ivonne Murillo-Rábago et al. in “Optimized Synthesis of Small and Stable Silver Nanoparticles Using Intracellular and Extracellular Components of Fungi: An Alternative for Bacterial Inhibition” used fungal extracts to create small-sized, stable silver nanoparticles, demonstrating their efficacy as antibacterial agents with potential clinical applications [11].
- Amal Adnan Ashour et al. in “Comparison and Advanced Antimicrobial Strategies of Silver and Copper Nanodrug-Loaded Glass Ionomer Cement against Dental Caries Microbes” show that adding metronidazole and copper nanoparticles extracted from *Thymus vulgaris* to glass ionomer cement increases the cement's antimicrobial efficacy against bacteria, while maintaining compressive strength and possibly enhancing dental restorations [12].
- Yolice Patricia Moreno Ruiz et al. in “Advanced Hydrogels Combined with Silver and Gold Nanoparticles against Antimicrobial Resistance” reviewed the potential of hydrogels combined with metallic nanoparticles as a promising approach to combat multidrug-resistant bacteria, emphasizing the antibacterial and antibiofilm properties, delivery mechanisms, and antimicrobial resistance [13].
- Michele Fiore et al. in “Is Silver the New Gold? A Systematic Review of the Preclinical Evidence of Its Use in Bone Substitutes as Antiseptic” reviewed the preclinical data of silver ions or silver nanoparticles in bone substitutes as antiseptic to bone infection treatments [14].

The contributions in this Special Issue highlight the significant potential of silver and gold compounds in combating bacterial infections. However, bringing these laboratory findings into clinical use will require collaboration among researchers, healthcare professionals, and policymakers. Ongoing research into innovative synthesis methods, deeper insights into their mechanisms of action, and comprehensive assessments of their safety and effectiveness are essential for progressing these compounds toward clinical applications.

We sincerely thank all the authors, reviewers, and the editorial team for their commitment and effort in making this Special Issue possible. We also express our gratitude to our readers for their interest and involvement in this important topic.

In light of the challenges posed by bacterial resistance and the ongoing search for new antibiotics, the research in this Special Issue provides a beacon of hope. The innovative strategies and encouraging outcomes showcased here emphasize the potential of silver and gold compounds as valuable tools in our antimicrobial arsenal. We encourage you to explore these articles, immerse yourself in the intriguing realm of antimicrobial metal compounds, and join us in the fight against bacterial infections to improve public health.
